# Utilization of NKX3.1 and P501S to distinguish primary breast carcinoma from metastatic prostatic adenocarcinoma in male patients

**DOI:** 10.1007/s00428-025-04124-3

**Published:** 2025-05-10

**Authors:** Mustafa Goksel, Chirag R. Patel, Sarah A. Anderson, Gian Piero Carames, Sandra S. Moultrie, Cristina Magi-Galluzzi, Shi Wei, Xiao Huang

**Affiliations:** 1 Department of Pathology, The University of Alabama at Birmingham, 619 19th Street South, Birmingham, AL 35294 USA; 2 Department of Laboratory Medicine and Pathology, Mayo Clinic, Phoenix, AZ 85054 USA

**Keywords:** NKX3.1, P501S, Breast carcinoma, Metastatic prostate cancer

## Abstract

*NKX3.1* is an androgen-regulated tumor suppressor gene, and NKX3.1 protein expression is present mainly in prostatic epithelium. P501S is expressed in prostatic epithelium and is also regulated by androgens. Immunohistochemistry (IHC) for NKX3.1 and P501S is commonly used for the diagnosis of metastatic prostate carcinoma in pathology practice. Male breast cancer is rare, and most of the tumors are androgen-receptor positive. Thus, we investigated NKX3.1 and P501S expression in male breast carcinoma to determine its role in distinguishing primary breast carcinoma from metastatic prostatic adenocarcinoma. IHC for NKX3.1 and P501S was performed on primary invasive breast carcinomas in 25 male patients, 5 of whom had prior history of prostatic adenocarcinoma. Immunoreactivity was classified as negative or positive staining. Expression of NKX3.1 and P501S was identified in 8 (32%) and 7 (28%) cases of invasive breast carcinomas, 42% and 20% of in situ breast carcinomas, and 8% and 46% of normal terminal ductal lobular unit (TDLU), respectively. Among the five patients with prior history of prostate carcinoma, four had NKX3.1 or P501S expression in their primary invasive breast carcinomas. Their diagnosis of primary invasive breast carcinomas was confirmed by GATA3 and estrogen receptor (ER) IHC. This is the first study to report NKX3.1 and P501S expression in primary breast carcinomas in male patients. The positive rates are higher than those reported in female patients with ductal carcinoma. When considering the use of IHC markers to confirm the diagnosis of primary breast carcinoma in male patients, breast-specific markers or hormonal receptors should be considered in the diagnostic panel.

**Supplementary Information:**

The online version contains supplementary material available at 10.1007/s00428-025-04124-3.

## Introduction

Prostate cancer is the most common non-dermatologic cancer in male patients in the USA [1]. Prostatic carcinoma is reportedly the most frequent primary tumor that metastasizes to the breast in male patients [2]. On the other hand, primary male breast cancer is rare and accounts for less than 1% of all breast cancers [3]. Distinguishing between primary breast carcinoma and metastatic prostate carcinoma solely by morphology may be challenging, especially in patients with a history of prostate cancer, where an in situ breast carcinoma component is absent. Immunohistochemistry (IHC) may be considered to differentiate primary breast cancer from metastatic prostate cancer.

*NKX3.1* is an androgen-regulated tumor suppressor gene associated with prostatic cancer progression [4, 5]. Although NKX3.1 has been shown to be an excellent marker for confirming prostatic origin in the metastatic setting, the protein is also expressed in other neoplastic and non-neoplastic tissues [6]. Variable NKX3.1 expression has been reported in primary breast carcinomas, ranging from 2 to 27% across different subtypes [4–8], mainly in estrogen receptor (ER)-positive and androgen receptor (AR)-positive tumors [4]. AR expression in male breast carcinomas (ranging from 81 to 95%) is higher compared to that in female patients (ranging from 21 to 80%) [9–14]. Therefore, we investigated NKX3.1 expression in male breast carcinomas.

P501S (Prostein, SLC45 A3) is predominantly expressed in prostatic epithelium and regulated by androgen [15]. The protein is highly sensitive for prostatic tissue, reportedly expressed in > 96% of prostatic carcinomas [16]. However, P501S expression has been seen in other tumor types, such as adenocarcinoma of the gastrointestinal tract, pancreas, and breast [16], hepatocellular carcinoma [17], urothelial carcinoma [18], and Sertoli-Leydig cell tumor [19]. To date, the diagnostic utility of distinguishing male breast carcinoma from metastatic prostatic cancer has not been reported. In addition, to the best of our knowledge, the NKX3.1 expression in male breast carcinomas has not yet been investigated.

## Materials and methods

### Study samples

The UAB pathology database was searched for primary male invasive breast carcinoma diagnosed from 2014 to 2024 to identify cases with biopsy and/or resection slides available for review and accompanying paraffin-embedded blocks available for IHC studies. Patient age at initial diagnosis, breast tumor size, and history of prostate cancer were collected from the slides review and the patients’ medical records after Institutional Review Board approval.

### Histology

All the original breast diagnostic slides were reviewed independently by two pathologists (XH and SAA) to confirm the pathologic tumor characteristics, including histologic subtype, histologic grade, and predictive marker (estrogen receptor (ER), progesterone receptor (PR), and human epidermal growth factor receptor 2 (HER2)) status, and to identify a representative paraffin block. The American Society of Clinical Oncology/College of American Pathologists guideline recommendations [20–22] were used to categorize ER, PR, and HER2 status as part of the routine pathologic evaluation. Tumors with ER-low positivity (1–10%) were considered ER-positive in this study.

### Immunohistochemistry for NKX3.1 and P501S

Immunohistochemical stains (IHC) were performed on 4-µm thick whole slide sections from representative formalin-fixed paraffin-embedded (FFPE) tissue using a rabbit monoclonal primary anti-NKX3.1 antibody (clone EP356, Ready-to-use, Cell Marque) and rabbit monoclonal primary anti-P501S antibody (clone EP381, Ready-to-use, Bio SB).

Both IHC stains for NKX3.1 and P501S were reviewed independently by a breast pathologist (XH) and a genitourinary pathologist (MG). Only NKX3.1 nuclear staining and P501S cytoplasmic granular staining in IHC were counted as positive. Immunoreactivity was classified as negative (scores 0–2) or positive (scores 3–4, 6, and 9) staining. The immunoreactivity scores were calculated by multiplying a percentage score of positive tumor cells (score 0, < 1%; score 1, 1–10%; score 2, 11–50%; score 3, 51–100%) by an intensity score (score 0, virtually complete absent; score 1, weak; score 2, moderate; score 3, strong).

For each breast tumor section, both IHC stains were evaluated in the invasive carcinoma component, non-invasive carcinoma component, and normal breast ducts. In this study, ductal carcinoma in situ (DCIS) (*n* = 12) and encapsulated papillary carcinoma (*n* = 4) (ER/PR-positive and HER2-negative) were considered non-invasive carcinoma.

## Results

### Clinicopathological features

A total of 25 patients who had available primary tumor specimens were included in this study. The clinicopathologic features are shown in Table 1. The mean age of patients was 69 years (range 42–93). Tumor size ranged from 2 to 40 mm. All tumors were invasive ductal carcinoma, NOS, 12 of which (48%) were Nottingham histologic grade 3, and 21 (84%) were ER/PR-positive and HER2-negative. Five (20%) patients had a known history of prostate cancer (Table 2).

**Table 1:** Clinicopathological features of invasive breast carcinoma in male patients

	Number of cases
Age at initial diagnosis (y)	
40–49	3
50–59	5
60–69	5
70–79	5
80–89	5
≥ 90	2
Tumor size (mm)	
≤ 1	0
1.1–20	11
20.1–50	13
> 50	0
Unknown	1
Diagnosis	
Invasive ductal carcinoma, NOS	25
Invasive lobular carcinoma	0
Nottingham histologic grade	
1	2
2	11
3	12
Hormonal receptor status	
ER/PR-positive HER2-negative	21
HER2-positive	3
TNBC	1
Known history of prostate cancer	
Yes	5
No	20

**Table 2:** Clinicopathologic features and immunohistochemistry of the primary breast carcinoma in the patients with history of prostate cancer

		Patient #1	Patient #2	Patient #3	Patient #4	Patient #5
History of prostate cancer						
Age at diagnosis of prostate cancer (y)		39	53	70	76	59
Subtype		Acinar	Acinar	Acinar	Acinar	Unknown
Gleason score (Grade Group)		3 + 3 = 6 (Grade Group 1)	3 + 4 = 7 (Grade Group 2)	3 + 4 = 7 (Grade Group 2)	4 + 4 = 8 (Grade Group 4)	Unknown
AJCC stage		IIA	IIB	IIB	IIC	Unknown
Treatment of prostate cancer		Surgery (unknown type) + Radiation	Radiation	Radical prostatectomy	Radiation + Androgen deprivation	Radical prostatectomy
Primary breast invasive carcinoma						
Age at diagnosis of breast cancer(y)		67	57	73	86	79
Subtype		IDC, NOS	IDC, NOS	IDC, NOS	IDC, NOS	IDC, NOS
In situ carcinoma component present		Yes	Yes	Yes	Yes	No
Nottingham histologic grade		3	3	3	3	3
Tumor size (mm)		14	30	15	40	7
Regional lymph node involvement		No	No	No	Yes	No
ER		Positive	Positive	Positive	Positive	Positive
PR		Positive	Positive	Positive	Positive	Positive
HER2		Negative	Negative	Negative	Negative	Negative
NKX3.1	Invasive carcinoma	Positive	Positive	Negative	Negative	Negative
	In situ component	Negative	Positive	Negative	N/A	N/A
	Normal	Negative	Negative	Negative	Negative	N/A
P501S	Invasive carcinoma	Negative	Negative	Negative	Positive	Positive
	In situ component	Negative	Positive	Negative	N/A	N/A
	Normal	Negative	N/A	Positive	Positive	Positive
AR	Invasive carcinoma	Positive	N/A	Positive	N/A	Positive
	In situ component	Positive	N/A	Positive	N/A	N/A
	Normal	Positive	N/A	Positive	N/A	Positive

### NKX3.1 and P501S expression in primary breast carcinoma

**Fig. 1:**
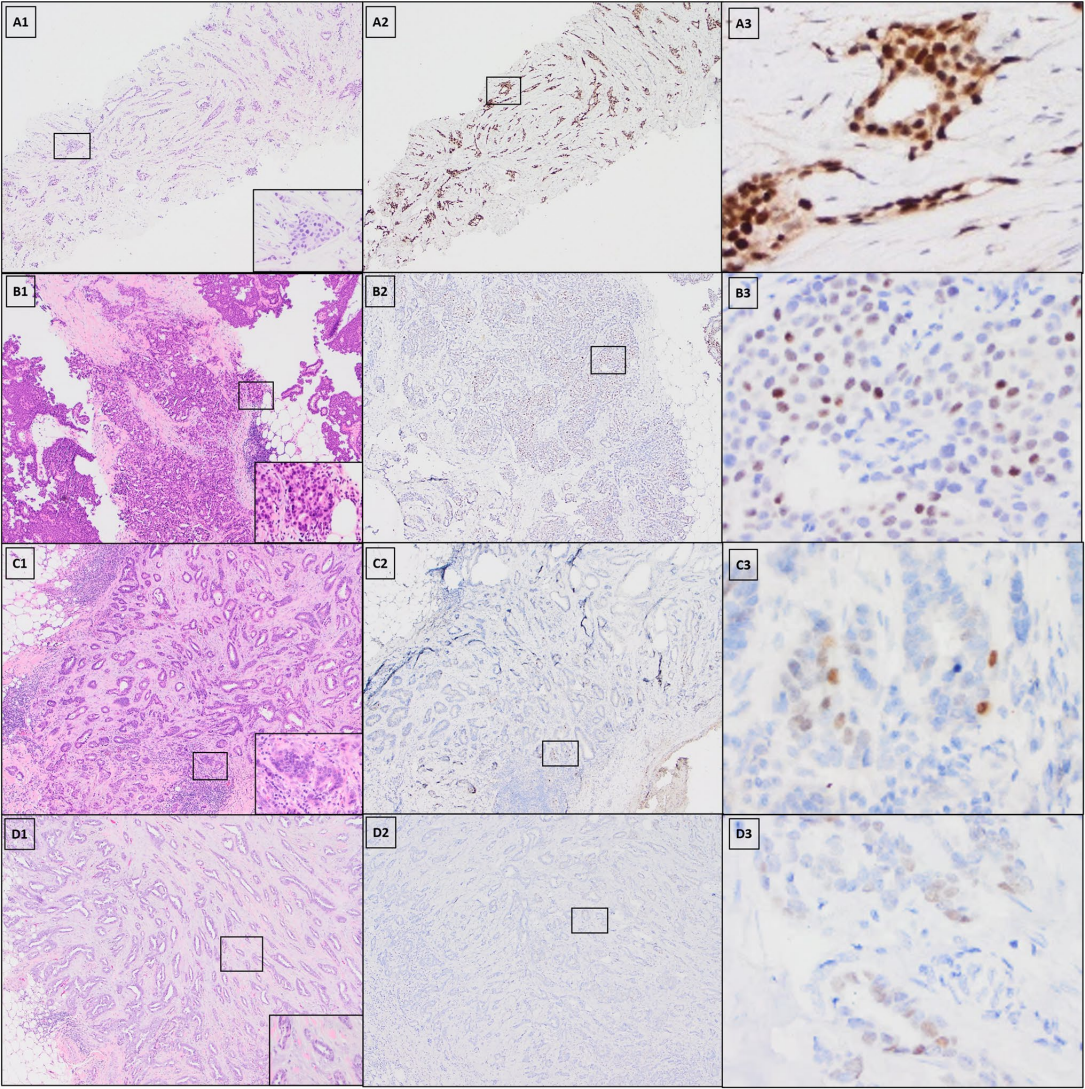
Expression of NKX3.1 in representative cases. A–D Each panel represents one invasive ductal carcinoma of breast. **A1**–**D1** Hematoxylin and eosin strain; **A2**–**D2** and **A3**–**D3** NKX3.1 stain (**A2** strong positivity; **B2** moderate positivity; **C2** low positivity; **D2** negative. **A2**–**D2** Original magnification, × 40; **A3**–**D3** higher-magnification images of the boxed areas, × 400)

**Fig. 2:**
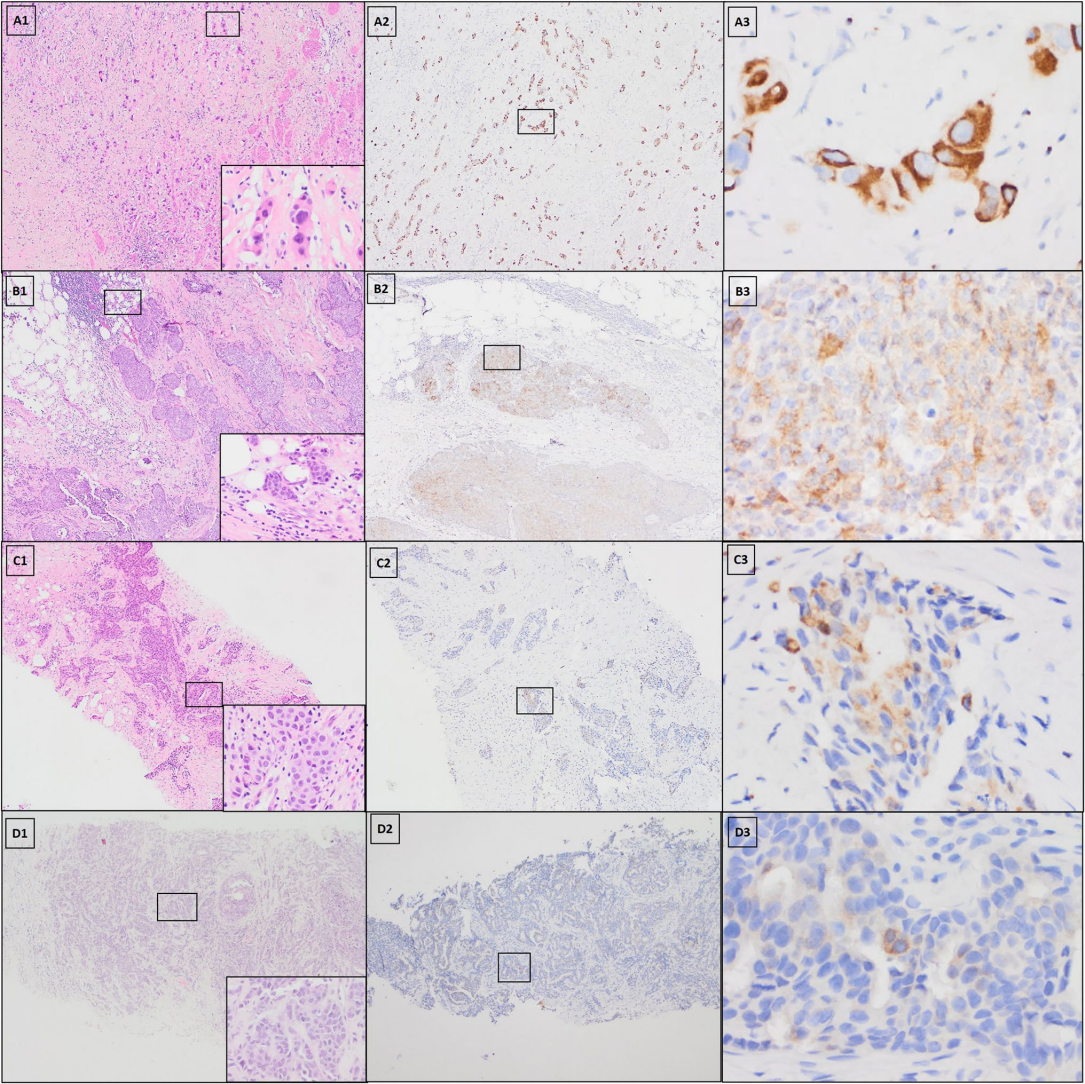
Expression of P501S in representative cases. A–D Each panel represents one invasive ductal carcinoma of breast. **A1**–**D1** Hematoxylin and eosin strain; **A2**–**D2** and **A3**–**D3** prostein stain (**A2** strong positivity; **B2** moderate positivity; **C2** low positivity; **D2** negative. **A2**–**D2** Original magnification, × 40; **A3**–**D3** higher-magnification images of the boxed areas, × 400)

**Fig. 3:**
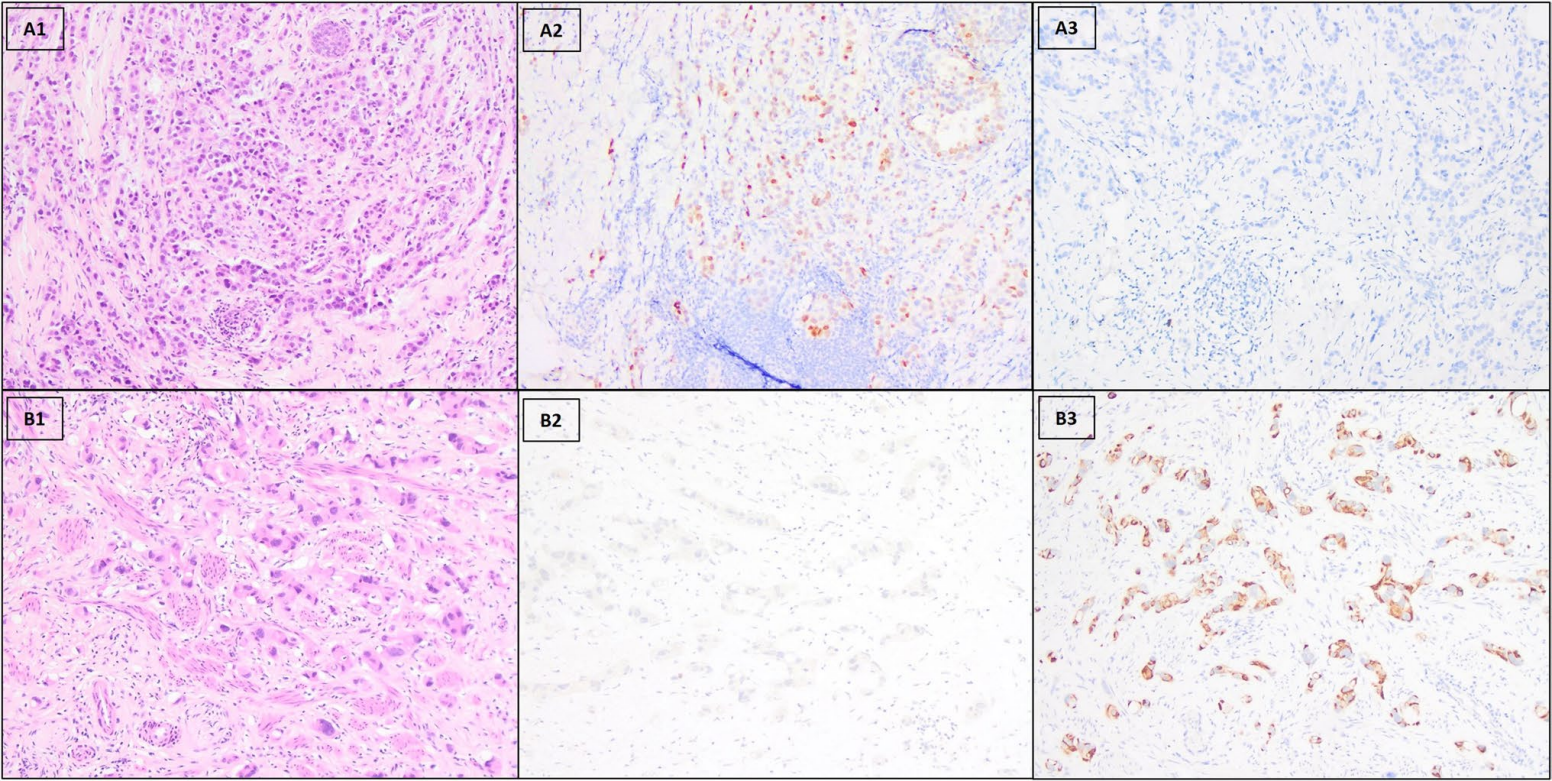
Representative cases with discordant expression of NKX3.1 and P501S. A. A primary breast carcinoma was positive for NKX3.1 (**A2**) and negative for P501S (**A3**). B. A primary breast carcinoma was negative for NKX3.1 (**B2**) and positive for P501S (**B3**). Original magnification, × 100

Expression of NKX3.1 and P501S in malignant and normal breast tissue is shown in Table 3. Representative images are shown in Figs. 1 and 2. Among the 25 invasive carcinomas, 8 (32%) were positive for NKX3.1, 5 of which were also positive for P501S, and 7 (28%) were positive for P501S, 3 of which were positive for NKX3.1. Concordance between NKX3.1 and P501S expression in invasive carcinoma is shown in Table 4. Among the 25 invasive tumors, 16 (64%) showed concordant expression. Thirteen cases (52%) were positive for both markers, and 3 (12%) were negative for both markers, respectively, as shown in Fig. 3.

**Table 3:** Expression of NKX3.1 and P501S in malignant and normal breast tissue

	NKX3.1			P501S		
	Negative	Positive	Total	Negative	Positive	Total
Invasive carcinoma	17	8	25	18	7	25
Non-invasive component	7	5	12	8	2	10
Normal mammary ducts	11	1	12	7	6	13

**Table 4:** Concordance between NKX3.1 and P501S expression in invasive carcinoma

		NKX3.1		Total (*n*)
		Negative (*n*)	Positive (*n*)	
P501S	Negative (*n*)	13	5	18
	Positive (*n*)	4	3	7
Total (*n*)		17	8	25

**Fig. 4:**
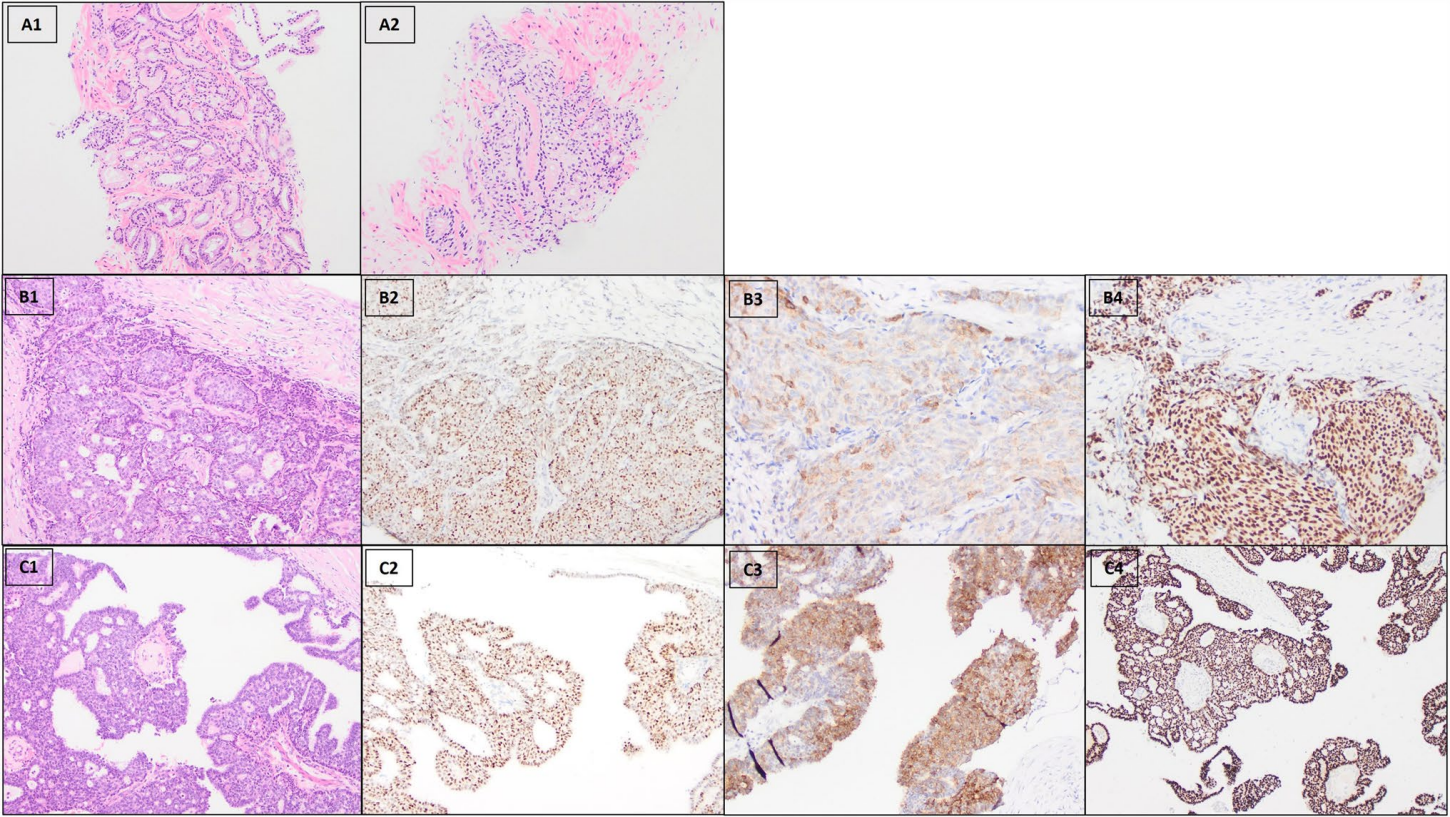
Invasive ductal carcinoma arising in an encapsulated papillary carcinoma from a male patient with history of prostatic adenocarcinoma. A. Prostate adenocarcinoma, Gleason score 3 + 4. B. NKX3.1 (**B2**), P501S (**B3**), and GATA3 (**B4**) staining in the invasive duct carcinoma of breast (**B1**). C. NKX3.1 (**C2**), P501S (**C3**), and GATA3 (**C4**) staining in the encapsulated papillary carcinoma of breast (**C1**). Original magnification, × 100

A total of 12 tumors (48%) had a non-invasive component in the NKX3.1-stained sections, 5 of which (42%) were positive. Of the 10 tumors with non-invasive components in the P501S-stained sections, 2 (20%) were positive. Four of the non-invasive tumors were encapsulated papillary carcinoma (EPC), which were all NKX3.1 positive (1 intermediate and 3 high expression), and two were P501S positive (high expression). In the invasive components of the 4 EPC, 3 were NKX3.1-positive (2 intermediate and 1 high expression), and one was P501S-positive (high expression). One patient with EPC had a history of prostate cancer; his primary breast carcinoma was confirmed by GATA3 and ER IHC. Selective examples are shown in Fig. 4.

**Fig. 5:**
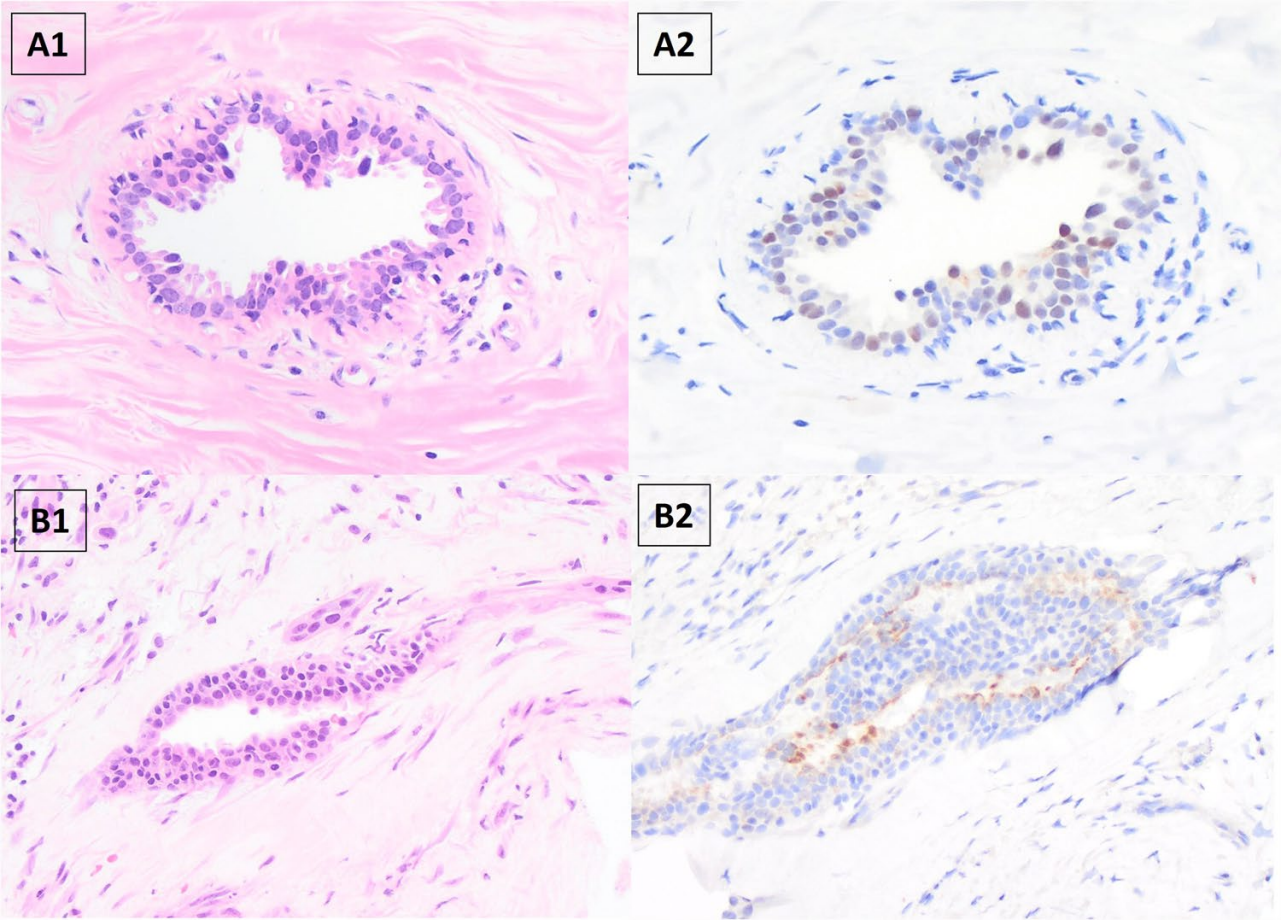
Representative cases of NKX3.1 and P501S expression in normal breast tissue. A. NKX3.1 staining in normal mammary duct (original magnification, × 200). B. P501S staining in normal mammary duct (original magnification, × 100)

One of twelve (8%) cases showed NKX3.1 moderate expression in the normal terminal ductal lobular units (TDLU), and the adjacent invasive carcinoma was negative for NKX3.1. Six of thirteen (46%) tumors showed P501S expression (2 intermediate and 4 high expression) in the normal TDLU, and 2 of them were P501S-positive in the adjacent invasive components. Examples are illustrated in Fig. 5.

### NKX3.1 and P501S expression of primary breast carcinoma in patients with history of prostate cancer

Five of the 25 patients had a known history of prostate cancer. The clinicopathologic features and IHC stains for these patients are summarized in Table 2. Two of the 5 invasive breast carcinomas in prostate cancer patients were positive for NKX3.1, and two were positive for P501S (Fig. 4). None of these cases were positive for both markers. All invasive breast carcinomas in prostate cancer patients were ER/PR positive and HER2 negative. Three cases showed P501S expression in normal breast ducts.

## Discussion

While male breast cancer is uncommon, the prevalence of prostate cancer is high. The latter is the most common origin for metastatic disease in male breast [1, 2]. In a subset of cases, immunohistochemistry is needed to distinguish primary breast cancer from metastatic prostate cancer. NKX3.1 IHC and P501S IHC stains are commonly used as organ-specific markers for the diagnosis of metastatic prostate cancer in pathology practice. We investigated NKX3.1 and P501S expression in breast carcinomas from 25 male patients.

Previous studies have shown variable NKX3.1 expression in female breast carcinomas, ranging from 2 to 75% (Table 5). One large cohort study showed an expression rate of 13% in triple-negative breast carcinoma (TNBC) [7]. It is of further interest that primary and metastatic breast carcinoma with lobular differentiation showed a higher NKX3.1 expression (up to 29%) than ductal tumors (up to 6%) [4, 6]. Two relatively larger cohorts showed 2% and 5% of NKX3.1 expression in invasive ductal carcinomas of the female breast, respectively [4, 6]. This is in contrast to 32% of NKX3.1 expression in our study, higher than the reported rates in the female invasive ductal carcinomas. It has been well reported that the vast majority of male breast carcinomas are AR-positive [9]. Given that NKX3.1 is an androgen-regulated tumor suppressor gene, it is not surprising that NKX3.1 expression is higher in male breast carcinomas than in female breast carcinomas. IHC methodologies, antibody clones, and sample size may also contribute to the variation in the literature.

**Table 5:** Previous reports of NKX3.1 and P501S immunohistochemistry in female breast lesions

Author	Case number	Lesions	NKX3.1		P501S	
			Clone/vendor/dilution	Positive	Antibody, clone	Positive
Han et al. [7]	631	TNBC	EP356/Roche/RTU	13% (85/631)	N/A	N/A
Kim et al. [8]	20	Prostatic metaplasia, gender-affirming	Polyclonal rabbit/Athena ES/1:1000	75% (15/20)	N/A	N/A
Gelmann et al. [6]	380	Metastatic breast carcinoma, ductal	Polyclonal [4]/1:1000	5% (19/380)	N/A	N/A
Gelmann et al. [6]	56	Metastatic breast carcinoma, lobular	Polyclonal [4]/1:1000	29% (16/56)	N/A	N/A
Asch-Kendrick et al. [4]	86	IDC	Polyclonal rabbit/Biocare/RTU	2% (2/86)	N/A	N/A
Asch-Kendrick et al. [4]	37	ILC	Polyclonal rabbit/Biocare/RTU	27% (10/37)	N/A	N/A
Gurel et al. [5]	18	Invasive breast carcinoma	Polyclonal rabbit/Athena ES/1:500	6% (1/18)	N/A	N/A
Viehweger et al. [16]	2305	Breast tumors	N/A	N/A	Rabbit recombinant monoclonal/MS Validated Antibodies/1:150	7% (163/2,305)
Mochizuki et al. [17]	30	IDC	N/A	N/A	10E3/Dako/1:400	3% (1/30)

*TNBC*, triple negative breast carcinoma; *RTU*, ready to use; *IDC*, invasive ductal carcinoma; *ILC*, invasive lobular carcinoma

An aforementioned study reported that P501S expression was detected in pilomatricoma and squamous cell carcinoma of the skin [16]. Very few studies have investigated P501S expression in female breast carcinomas. An early study found P501S immunoreactivity in 1 of 30 (3%) invasive ductal breast carcinomas in female patients [17], much lower than the 28% in our study. This could be a result of gender disparity, antibody clones, or IHC techniques, thus warranting further investigation. In a large cohort, P501S expression was found in 7% (163/2305) of breast cancers. Interestingly, detectable P501S expression was associated with histologic grade 3, HER2 positivity, and ER negativity in invasive ductal breast carcinomas [16]. In the present study, all 7 P501S-positive tumors were ER-positive, one was HER2-positive, and four were histologic grade 3. Of the 18 P501S-negative tumors, one was ER-negative, two were HER2-positive, and eight were histologic grade 3. Our study failed to show an association between P501S expression and pathological features in male breast carcinomas.

NKX3.1 and P501S expression have not been reported in normal breast tissue and in in situ breast carcinoma in previous studies [6, 16]. We thus looked into the expression of these two markers in in situ carcinoma and normal breast tissue. NKX3.1 and P501S expression in the normal TDLU was found in 8% and 46% of cases, respectively. Furthermore, 42% and 20% of cases showed NKX3.1 and P501S expression, respectively, in in situ carcinoma components. Conversely, P501S expression was seen in all 5 cases in which sweat glands were present in the staining sections (Supplemental Figure 1). Thus, “prostatic-specific” markers were detected in at least a subset of in situ carcinomas, normal breast elements, and skin adnexal structures in male patients.

Papillary carcinoma of the breast represents about 0.5% of all breast cancers [23]. It remains rare in men and accounts for 5–7.5% of all male breast carcinomas [24]. Among the prostatic tumors, ductal adenocarcinoma can metastasize and show similar morphologic features to EPC. In our study, 12 cases had in situ carcinoma components, including 4 EPC and 8 conventional ductal carcinoma in situ (DCIS). NKX3.1 expression was found in all EPCs and 1 (13%) conventional DCIS, respectively. P501S immunoreactivity was found in 2 (50%) EPCs, but not in conventional DCIS. In the invasive components of the 12 cases, 3 (75%) from EPC were NKX3.1-positive and 2 (25%) from conventional DCIS, respectively. One case of invasive carcinoma (25%) arising from EPC was P501S-positive. This is in contrast to 1 case (13%) of invasive carcinomas derived from conventional DCIS. Thus, papillary carcinomas showed higher expression of prostatic markers than conventional DCIS. Solely prostatic markers are not reliable to distinguish EPC from metastatic ductal adenocarcinoma of the prostate.

Synchronous or metachronous co-occurrences of prostate cancer and breast cancer in male patients have been reported. Leibowitz et al. reported 10 (6%) of 161 male patients with breast cancer concurrently diagnosed with prostate cancer between 1977 and 2000 at Dana-Farber Cancer Institute and Massachusetts General Hospital [25]. Lee et al. reported that 12 (17%) of 69 male patients with breast cancer, who had a diagnosis of prostate cancer between 1990 to 2006 at the Cleveland Clinic [26]. In our study, five (20%) breast cancer patients had a prior diagnosis of prostate cancer. Invasive breast carcinomas from 4 (80%) patients showed expression of prostate markers. On the contrary, among the 20 patients without known history of prostate cancer, 8 (40%) breast tumors showed expression of prostate markers. Breast carcinomas in patients with history of prostate cancer seem to have a higher rate of prostate marker expression in this limited study. Additionally, concordance of NKX3.1 and P501S expression was only found in 64% of cases in our study. Therefore, distinguishing between breast and prostate cancers in this subset of patients should not solely rely on a single prostate marker.

## Conclusion

Our study demonstrated expression of NKX3.1 and P501S in primary breast carcinomas in male patients. In cases with equivocal morphological features, lack of an in situ carcinoma component, and history of prostate cancer, breast markers may be considered for the diagnosis of primary breast cancer. In the scenario of evaluating distant metastasis in patients with late-stage diseases, an immunohistochemistry panel to include both breast and prostatic markers should be considered.

## Supplementary Information

Below is the link to the electronic supplementary material.

Supp. Figure 1. P501S staining in normal sweat glands of skin (A1 and A2, Original magnification: × 100; A3, Boxed area, Original magnification: × 400). (JPG 741 KB)

## Data Availability

All data generated or analyzed during this study are included in this article. Further inquiries can be directed to the corresponding author.
