# Supplementary figures and images for: Utilization of NKX3.1 and P501S to distinguish primary breast carcinoma from metastatic prostatic adenocarcinoma in male patients
Source: Virchows Arch. 2025 May 10;488(2):377–86. doi: 10.1007/s00428-025-04124-3 (PMC12916889; doi:10.1007/s00428-025-04124-3)

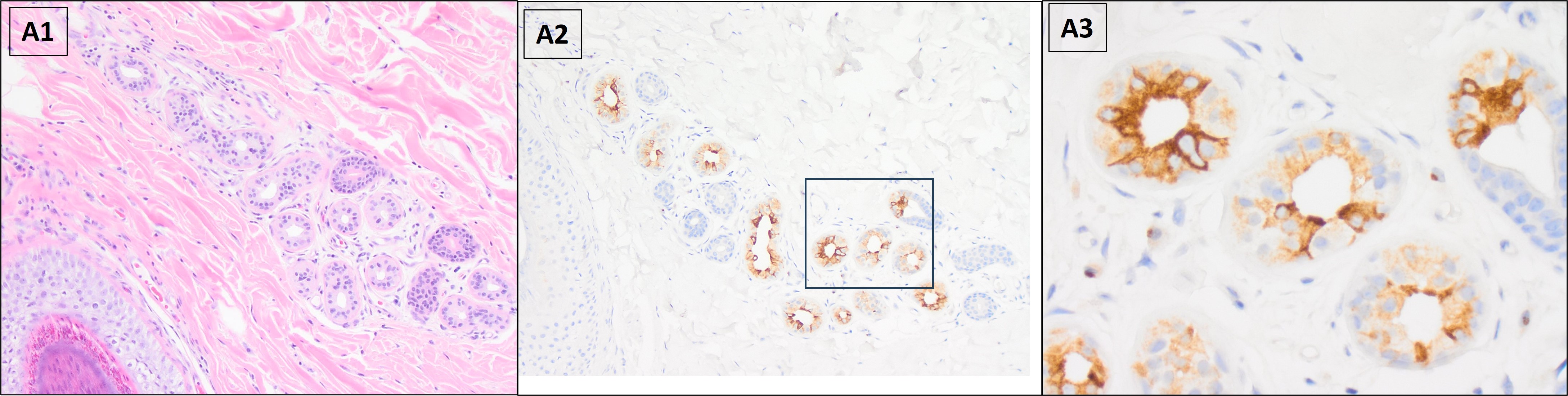

Supplement: Supplementary file 1 — Supp. Figure 1. P501S staining in normal sweat glands of skin (A1 and A2, Original magnification: × 100; A3, Boxed area, Original magnification: × 400). (JPG 741 KB) [file 428_2025_4124_MOESM1_ESM.jpg]
